# The Role of B Cell and T Cell Glycosylation in Systemic Lupus Erythematosus

**DOI:** 10.3390/ijms24010863

**Published:** 2023-01-03

**Authors:** Ivan Ramos-Martínez, Edgar Ramos-Martínez, Marco Cerbón, Armando Pérez-Torres, Laura Pérez-Campos Mayoral, María Teresa Hernández-Huerta, Margarito Martínez-Cruz, Alma Dolores Pérez-Santiago, Marco Antonio Sánchez-Medina, Iván Antonio García-Montalvo, Edgar Zenteno, Carlos Alberto Matias-Cervantes, Víctor Ojeda-Meixueiro, Eduardo Pérez-Campos

**Affiliations:** 1 Departamento de Medicina y Zootecnia de Cerdos, Facultad de Medicina Veterinaria y Zootecnia, Universidad Nacional Autónoma de México, Ciudad de México 04510, Mexico; 2 Facultad de Química, Universidad Nacional Autónoma de México, Ciudad de México 04510, Mexico; 3 Escuela de Ciencias, Universidad Autónoma Benito Juárez de Oaxaca, Oaxaca 68120, Mexico; 4 Unidad de Investigación en Reproducción Humana, Instituto Nacional de Perinatología “Isidro Espinosa de los Reyes”—Facultad de Química, Universidad Nacional Autónoma de México, Ciudad de México 04510, Mexico; 5 Departamento de Biología Celular y Tisular, Facultad de Medicina, Universidad Nacional Autónoma de México, Ciudad de México 04510, Mexico; 6 Facultad de Medicina, Universidad Autónoma Benito Juárez de Oaxaca, Oaxaca 68120, Mexico; 7 CONACyT, Facultad de Medicina y Cirugía, Universidad Autónoma “Benito Juárez” de Oaxaca (UABJO), Oaxaca 68020, Mexico; 8 Tecnológico Nacional de México/IT Oaxaca, Oaxaca 68030, Mexico; 9 Departamento de Bioquímica, Facultad de Medicina, Universidad Nacional Autónoma de México, Ciudad de México 04510, Mexico

**Keywords:** autoimmune diseases, glycans, post-translational modifications, lectins, immunoglobulins, T cells, B cells

## Abstract

Glycosylation is a post-translational modification that affects the stability, structure, antigenicity and charge of proteins. In the immune system, glycosylation is involved in the regulation of ligand–receptor interactions, such as in B-cell and T-cell activating receptors. Alterations in glycosylation have been described in several autoimmune diseases, such as systemic lupus erythematosus (SLE), in which alterations have been found mainly in the glycosylation of B lymphocytes, T lymphocytes and immunoglobulins. In immunoglobulin G of lupus patients, a decrease in galactosylation, sialylation, and nucleotide fucose, as well as an increase in the *N*-acetylglucosamine bisector, are observed. These changes in glycoisolation affect the interactions of immunoglobulins with Fc receptors and are associated with pericarditis, proteinuria, nephritis, and the presence of antinuclear antibodies. In T cells, alterations have been described in the glycosylation of receptors involved in activation, such as the T cell receptor; these changes affect the affinity with their ligands and modulate the binding to endogenous lectins such as galectins. In T cells from lupus patients, a decrease in galectin 1 binding is observed, which could favor activation and reduce apoptosis. Furthermore, these alterations in glycosylation correlate with disease activity and clinical manifestations, and thus have potential use as biomarkers. In this review, we summarize findings on glycosylation alterations in SLE and how they relate to immune system defects and their clinical manifestations.

## 1. Introduction

Glycosylation is a very common post-translational modification; it is present in virtually all secreted proteins and membrane proteins. It plays a key role in folding and transport through the endoplasmic reticulum and the Golgi apparatus [1,2]. Once the protein has completed its synthesis and transport phase, additional glycosylation can affect its stability, function, charge, and antigenicity. Glycosylation modifications can occur for a single monosaccharide or dozens of monosaccharides arranged in various three-dimensional structures [3].

Glycosylation is a mechanism that regulates many immune system processes, such as ligand–receptor interaction [4]. Changes in the glycosylation of the T cell receptor complex or its co-receptors affect the activation threshold of T cells by modifying the affinity for their ligands [5]. In the case of immunoglobulins, it has been reported that changes in glycosylation of heavy chain constant regions may be the difference between a pro-inflammatory or anti-inflammatory response [6]. Furthermore, glycosylation in cells of the immune system can be recognized by endogenous lectins such as galectins, selectins, and dectins that mediate functions such as cell activation, differentiation, and survival [4,7]. Therefore, alterations in glycosylation could lead to the development of autoimmune diseases.

Glycosylation abnormalities have been reported in autoimmune diseases such as IgA nephropathy, rheumatoid arthritis, inflammatory bowel disease, and multiple sclerosis [8,9]. For example, galactose deficiency in immunoglobulin G (IgG) in patients with rheumatoid arthritis correlates with disease progression [10]. Galactose deficiency in immunoglobulin G has also been reported in other diseases, such as inflammatory bowel disease [11] and multiple sclerosis [12]. In addition, decreased immunoglobulin G fucosylation is a marker of autoimmune thyroid diseases [8].

**Figure 1:**
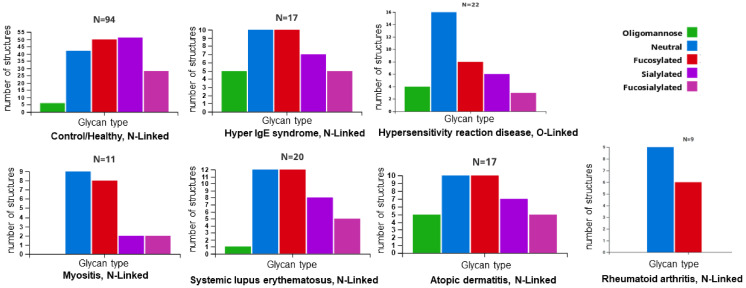
Compositional profiles of glycosylations related to diseases with alterations of the immune system. The number of glycans that have been associated with the indicated diseases is shown; in addition, the graphs show the frequency of sugars in the disease-related glycans. Neutral: does not contain sialic acid (*N*-acetylneuraminic acid [NeuAc] or *N*-glycolylneuraminic acid [NeuGc]), nor sulfate (Su).

In this review, we have observed the compositional profiles of *N*-glycans in various diseases in which the immune system plays an important role. These profiles were obtained with the GlyConnect Compozitor database [13], which identifies the glycans that have been related to some diseases and plots how many structures of certain sugars have been observed in these glycosylations (Figure 1). We can note that the composition of *N*-glycans is altered in several diseases, including SLE, compared to healthy individuals. A common alteration in these diseases is the decrease in sialic acid in *N*-glycans.

Regarding SLE, several reports identify changes in protein glycosylation, mainly in T cells and B cells. Here, we summarize the findings on glycosylation alterations in SLE and how these alterations relate to alterations in immune system homeostasis and clinical manifestations. Furthermore, we review findings in murine models where impaired glycan synthesis leads to the development of lupus-like diseases.

## 2. Systemic Lupus Erythematosus

Systemic lupus erythematosus (SLE) is an autoimmune disease that can affect every organ and system in the body. It presents a wide spectrum of clinical manifestations in acute periods and periods of remission. In mild forms, skin and joint manifestations are observed; in severe forms, it affects the kidneys, heart, and nervous system [14]. SLE develops mainly in women of reproductive age, in a ratio of nine women to one man [15].

Etiopathogenesis of SLE involves immunological, genetic, hormonal, and environmental factors that play an important role in the regulation of immunological tolerance [14,16]. B cells and their various subpopulations play a crucial role in the pathogenesis of SLE. These cells show abnormal activation through their B cell receptor and Toll-like receptors [17]. Another factor that favors the activation of B cells in SLE is altered levels of cytokines such as interferons alpha (IFNɑ), beta (IFNβ), and gamma (IFNɣ), as well as BAFF/Blys, which promote B cells survival [17,18].

Serum of SLE patients is characterized by having autoantibodies against antigens such as double-stranded DNA (dsDNA), single-stranded DNA (ssDNA), RNA, ribonucleoproteins (rRNA/Ro, La, Smith), and cytoskeletal antigens [19]. The autoantibodies in SLE patients are of a high affinity and IgG type, indicating that an isotype switch has occurred, for which the participation of T cells is required [20]. T cells from SLE patients overexpress the CD40L molecule, which allows them to provide costimulatory signals necessary for B cell differentiation, proliferation, and isotype switching [21].

The fundamental role of T cells in the etiopathogenesis of SLE is supported by studies in murine models, where mice develop lupus spontaneously. While in NZB/NZW F1(B/W) mice, T cells elimination by monoclonal antibodies prevented disease development [22]; as well as the fact that the hybrid nu/nu NZB/NZW F1 (B/W) mice, which lack a thymus, do not develop lupus [23].

For more information on the role of B cells and T cells in SLE, we recommend the following reviews [17,24,25].

## 3. Glycosylation

Glycosylation is the process by which sugars are added to proteins and lipids; this is carried out in specialized cell compartments such as the rough endoplasmic reticulum and the Golgi apparatus. In eukaryotic cells, we have two main types of glycosylation: *N*-glycosylation and *O*-glycosylation. *N*-glycosylation begins in the rough endoplasmic reticulum and is characterized by the binding of *N*-acetylglucosamine (GlcNAc) to asparagine residues in asparagine–X–threonine consensus sequences. *O*-glycosylation takes place primarily in the Golgi apparatus and is characterized by the binding of *N*-acetylgalactosamine (GalNAc) to serine or threonine residues in proline-containing consensus regions [1,2].

### 3.1. O-Glycosylation

**Figure 2:**
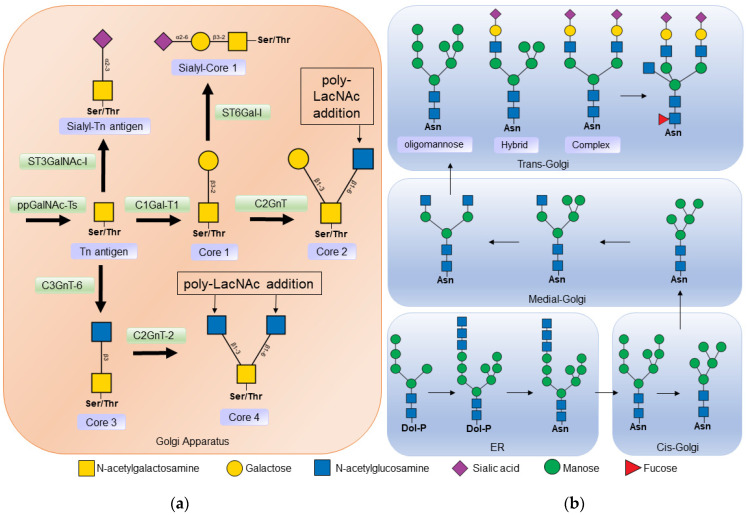
(**a**) Biosynthesis of *O*-glycans. *O*-glycosylation occurs in the Golgi apparatus, where *N*-acetyl-galactosamine is linked to a serine or threonine residue by the action of polypeptide *N*-acetylgalactosaminyltransferase (ppGalNAc-Ts) forming the Tn antigen. Core 1 β1-3 galactosyltransferase 1 (C1Gal-T1) then binds to a galactose to form core 1. Core 1 can be a substrate for some sialyltransferase or core 2 β1-6 *N*-acetylglucosaminyltransferase (C2GnT) to thus form core 2. Core 3 is formed from insufficient Tn by the action of core 3 β1-3 *N*-acetylglucosaminyltransferase 6 (C3GnT-6); in turn, core 3 is a substrate to form core 4 by the addition of another N -acetyl-glucosamine by Core 2/4 β1-6 *N*-acetylglucosaminyltransferase 2 (C2GnT-2). (**b**) Biosynthesis of *N*-glycans. Synthesis begins in the endoplasmic reticulum (ER), where a dolicholphosphate oligosaccharide is transferred to an asparagine residue by oligosaccharyltransferase (OST). Next, α-glucosidases I and II, and α-mannosidase I cleave the sugars from the oligosaccharide. The protein is then transported to the Golgi apparatus, where other mannosidase result from the processing of the *N*-glycan. In the middle part of the Golgi apparatus, *N*-acetylglucosaminyltransferase 1 (MGAT1) adds *N*-acetyl-glucosamine to begin the synthesis of hybrid and complex *N*-glycans. In the Trans Golgi part, fucoses, sialic acids, and poly-lactosamine chains, α2-6 sialyltransferase (ST3GalNAc), α2-6 sialyltransferase (ST6Gal), poly-*N*-acetyllactosamine (Poly-LacNAc) are added.

In *O*-glycosylation, *N*-acetyl-galactosamine (GalNAc) residues are added from *N*-acetyl-galactosamine-uridine diphosphate (UDP-GalNAc) by the action of *N*-acetyl-galactosaminyltransferase (ppGalNAcT). At least 21 isoforms of this enzyme have been described, which have different affinities depending on the amino acid sequence and the three-dimensional arrangement of carbohydrate residues [26,27]. The binding of *N*-acetyl-galactosamine (GalNAc) to the serine or threonine of a protein is known as the Tn antigen. In a second step, galactose (Gal) is added to the *N*-acetyl-galactosamine residue in the β1-3 bond, by the action of Core 1 β1-3 galactosyltransferase (C1GalT-1), to form the structure known as core 1 (Figure 2a) [2,28].

Core 1 may be a substrate for the enzyme core 2 β1-6 *N*-acetylglucosaminyltransferase (C2GnT), which catalyzes the addition of *N*-acetylglucosamine (GlcNAc) at the β1-6 bond with galactose [28]. Two isoforms of this enzyme have been described: the L type that synthesizes core 2, and the M type that also participates in the synthesis of other cores [2,29]. Core 2 is produced at many sites, including the intestinal mucosa, on activated T cells, and during embryonic development. The synthesis of cores 3 and 4 is mainly restricted to sites such as the mucosal epithelium of the gastrointestinal tract, the respiratory tract, and the salivary glands [2,29].

Core 1 can also be a substrate for sialyltransferases, which can bind sialic acid to carbon 3 of galactose and carbon 6 of *N*-acetyl-galactosamine [2]. Since sialic acid is negatively charged, its binding to receptors regulates ligand–receptor interaction by changing the charge of the molecule, and prevents further modifications of core 1 [30]. Between core 2 β1-6 *N*-acetylglucosaminyltransferase (C2GnT) and sialyltransferases, there is competition for core 1, and thus an increase in the activity of α3-sialyltransferase prevents further glycosylation of core 1 [31].

### 3.2. N-Glycosylation

The first sugar to be attached to eukaryotic *N*-glycans is *N*-acetyl-glucosamine, which is linked to the asparagine residue via an *N*-glycosidic bond in the consensus sequence Asn-X-Ser/Thr, where X is any amino acid except proline. *N*-glycans affect the conformation, solubility, antigenicity, activity, and recognition of proteins [1].

*N*-glycans have a common core sequence and are classified into three types based on the branches they contain. Oligomannose types contain only mannose residues in the branches, complex types have *N*-acetyl-glucosamine-initiated branches, and hybrid types have both mannose-initiated and *N*-acetyl-glucosamine-initiated branches (Figure 2b). *N*-acetyl-glucosamine-initiated branches can be lengthened by the addition of *N*-acetyl-lactosamine (Galβ1-4GlcNAc) repeats, which are ligands for endogenous lectins such as galectins [1,32].

The synthesis of *N*-glycans begins in the membrane of the endoplasmic reticulum with the addition of 14 sugars to the lipid molecule dolichol-phosphate. Then this oligosaccharide is transferred by oligosaccharyltransferase (OST) to an asparagine protein. In the lumen of the endoplasmic reticulum, the 14-sugar oligosaccharide is cleaved by α-glucosidase I (MOGS) and α-glucosidase II (GANAB) [1]. This step is important in protein folding, as chaperones in the endoplasmic reticulum recognize the cleaved *N*-glycans [33]. Glycosyltransferases and glucosidases are differentially expressed, and their activity is regulated by metabolism and physiological conditions, making them a control point for *N*-glycosylation [34].

Before leaving the endoplasmic reticulum, α-mannosidase I (MAN1B1) removes the α1-2 terminal mannose from the central arm of the *N*-glycan and is then transported to the *cis*-Golgi region, where other α-mannosidases such as α1-2 mannosidases IA (MAN1A1), IB (MAN1A2) and IC (MAN1C1) continue *N*-glycan processing.

The synthesis of hybrid and complex *N*-glycans begins in the medial part of the Golgi apparatus. *N*-acetylglucosaminyltransferase 1 (MGAT1) adds *N*-acetylglucosamine residues to specific mannoses in the *N*-glycan core, then α-mannosidase II (MAN2A1 or MAN2A2) removes other mannose residues to form the biantennary *N*-glycans that are produced by the action of *N*-acetylglucosaminyltransferase 2 (MGAT2). Hybrid *N*-glycans are formed if α-mannosidase II does not act on the glycan produced by MGAT1, or due to the incomplete action of α-mannosidase II. Most hybrid and complex *N*-glycans exhibit extended branching with the addition of *N*-acetylactosamine [1].

Finally, other sugars, such as sialic acid and fucose, as well as sulfate, are added to the branches. Sialic acids can be linked to *N*-acetyl-glucosamine through α-2,3 or α-2,6 linkages. These links are generated by sialyltransferases, enzymes present mainly in the trans part of the Golgi apparatus. The differential expression of sialyltransferases generates variability in the sialoglycans present in specific tissues; this difference not only occurs between organisms and cells of different tissues, but also varies in time, space, and environmental cues. In the case of fucose, it is added to the *N*-acetyl glucosamine linked to an asparagine by the action of α1-6 fucosyltransferase (FUT8) [35].

## 4. Functions of Glycosylation in the Immune System

Glycosylation affects the conformation, stability, charge, protease resistance, and affinity of the proteins for other proteins [3]; that is, it is part of the regulation of ligand–receptor interactions. During the inflammation process, the receptor proteins involved in migration, adhesion, and diapedesis are differentially glycosylated, thus they are recognized by their ligands [3]. For example, the molecule GlyCAM-1 (glycosylated cell adhesion molecules) expresses sulfur-containing *O*-glycans that bind L-selectin and thus regulate leukocyte migration to mesenteric and peripheral lymph nodes [36].

In the case of T cells, protein glycosylation is a dynamic process and changes depending on the stimuli they receive; for example, the pattern of glycosylation between naïve T cells and active T cells is different [37]. When the T cell is activated by stimuli through its T cell receptor (TCR), an increase in Tn antigen on glycoproteins is observed, as recognition of these cells by *Arachis hypogaea* agglutinin (PNA) and *Helix pomatia* agglutinin, two lectins with an affinity for the Tn antigen, is increased [38,39,40].

Glycosylation also regulates T cell activation and maturation by affecting the affinity between the T cell receptor complex and the major histocompatibility complex (MHC). Glycosylation of membrane receptors such as CD4, CD8, and T cell receptors has been shown to alter the affinity of interactions with the MHC, thereby affecting positive selection (Zhou et al., 2014). In addition, the presence of sialic acid in position α2-3 in core 1 of the O-glycans of the CD8 co-receptor decreases the avidity for MHC class I molecules, which plays an important role in negative selection. Glycosylation is also involved in the control of T cell differentiation. Decreased CD25 glycosylation has been shown to prevent T cell differentiation to the Th1, Th2, and Treg-induced phenotype, but promotes Th17 cell differentiation [41].

Glycoprotein sialylation is another important factor that regulates the interaction between receptors and their ligands. The interaction between CD69 and the S100A8/S100A9 complex is important in Treg cell differentiation, but the removal of sialic acid by sialidase treatment on activated CD4 T cells prevents binding between these proteins and decreases Treg cells differentiation [42].

Therefore, alterations in glycosylation lead to changes in the T cell-mediated immune response and have been linked to inflammation and autoimmune diseases [9,43]. For example, treatment of mice with glucosamine decreases CD25 glycosylation, prevents differentiation to Th1 cells, increases islet graft survival in diabetic mice, and exacerbates the severity of experimental autoimmune encephalitis [41].

In this regard, it has been shown that naïve, memory, and germinal center B cells express triantennary and tetraantennary *N*-glycans with long chains of poly-*N*-acetyl-lactosamine; these chains show specific differences that allow them to be ligands for certain galectins [44]. Galectins are lectins secreted by different cells, such as endothelial cells, monocytes, and dendritic cells, which recognize lactosamine in *O-* and *N*-glycans expressed in membrane proteins. The binding of galectins to their ligands triggers signals related to inflammation, activation, and death of target cells [45].

In B cells, the interaction of glycans and galectins regulates processes such as activation (galectin 3 and 9), plasma cell differentiation (galectin 1 and 8), and survival (galectin 1) [7]. Alterations in glycosylation have effects on the affinity for galectins. For example, in germinal center B cells, it has been observed that the addition of the disaccharide β1,6 *N*-Acetyl-glucosamine-galactose to the poly-*N*-acetyl-lactosamine chains prevents the binding of galectin 3 and 9 [44,46]. On the other hand, the addition of sialic acid at the α2,6 bond prevents the binding of galectin 1 [47].

In B-cell chronic lymphocytic leukemia, B-cell glycosylation is altered in a way that allows interaction with galectin 1, thereby lowering the B cell receptor (BCR) signaling threshold; this causes the signaling and survival of these B cells; in addition, the interaction of galectin 1 with glycans stimulates the expression of BAFF and APRIL that promote cell survival [48].

In T cells, galectins also regulate processes such as activation, differentiation, and apoptosis. Galectin 1 has been reported to stimulate T cell apoptosis through the recognition of CD43 [49]. Another ligand for galectin 1 is the glycoprotein CD45, which must express core 2 to trigger apoptotic signals [50]. To further explore the role of galectins and their function in B and T cells, we recommend the following reviews [7,51,52].

Likewise, immunoglobulins G (IgG) contain glycans in their heavy chain constant region-2 (CH2) of the Fc fragment. The heterogeneity of glycans in the Fc region is a mechanism that regulates their binding to Fc and C1q receptors [53]. Glycans lacking terminal galactoses in IgGs activate, complement, and promote proinflammatory reactions, whereas the addition of sialic acids to IgG glycans promotes anti-inflammatory effects [6].

Glycans are involved in the regulation of various processes related to the immune system and alterations in glycosylation can lead to the development of autoimmune diseases [9]. Some of the mechanisms that cause alterations in glycosylation include changes in the expression levels of glycosyltransferases, altered localization of enzymes within subcellular compartments, or changes in the enzymatic activity of glycosyltransferases [54,55].

## 5. Alterations in Glycosylation in Systemic Lupus Erythematosus

The first studies showing glycosylation alterations in SLE used lectins to determine glycan changes in T cells and immunoglobulins [56,57,58]. Subsequently, mass spectrometry techniques allowed the characterization of a large number of glycans in the membrane proteins of SLE patients [59,60], and molecular biology techniques have made it possible to identify changes in the expression of the enzymes involved in their synthesis [59]. Many of these alterations in glycosylation have been related to the regulation of the immune system and the development of clinical manifestations [61,62] e.g., in murine models with alterations in glycan synthesis, the development of lupus-like diseases has been observed.

Likewise, in the case of mice lacking the enzyme α-mannosidase II, they develop a disease similar to lupus [63]. The absence of α-mannosidase II results in the lack of complex branched *N*-glycans [64]. In these mice, an increase in the production of serum immunoglobulins, the development of lupus nephritis, and antinuclear antibodies against histones, Smith antigen, and DNA are detected [63]. It has also been observed that these have fewer fucosylated and galactosylated complex glycans in the kidney, but express a greater number of mannose-terminated oligosaccharides [65].

The damage suffered by mice lacking α-mannosidase II is mediated by immune system cells, such as macrophages that express the macrophage mannose receptor (MMR) and mannose-binding lectins (MBL-A), through which are recognized mannose-enriched glycans that have been exposed for lack of α-mannosidase II [66]. Mice also showed macrophage infiltration in the kidney; these macrophages express activation markers such as MHCII, and their number correlates with the degree of inflammation and tissue damage [66].

Similarly, kidney biopsies from patients with lupus nephritis showed an increase in mannose-rich glycans and a decrease in complex *N*-glycans compared with samples from healthy individuals. The increase in mannosylation was related to a decrease in the enzyme α-mannosidase II (MAN2A1), which causes an incomplete *N*-glycosylation pathway, and an increase in *O*-mannosyltransferase (POMT1), increasing masonylation of *O*-glycans. In addition, increased recognition of *Galanthus nivalis* agglutinin, which binds highly mannosylated glycans, was observed; this increase was associated with an increased risk of developing chronic kidney disease [59]. The increase in mannose residues is a marker of immunogenicity since they can be recognized by antigen-presenting cells through receptors such as DC-SIGN [67].

Therefore, similar results have been observed in terms of alterations in glycosylation in murine models and SLE patients. Most of the reports have focused on the study of T cells and B cells, but few are reports on other cells of the immune system or other systems. Similarly, changes in immunoglobulin glycosylation have been extensively characterized in SLE patients.

### 5.1. Alterations in B Cell Glycosylation and Antibodies in SLE

In the case of B cells, glycosylation studies have been directed primarily at immunoglobulins. It has been proposed that the acquisition of *N*-glycosylation sites in the variable regions of immunoglobulin light or heavy chains could favor B cell survival during germinal center selection, providing B cells with an additional mechanism for the generation of autoreactive cells [68,69].

#### 5.1.1. Glycosylation of Constant Regions

SLE patients have alterations in the glycosylation of immunoglobulin G. A greater recognition of the *Aleuria aurantia* lectin, which has an affinity for fucosylated residues, has been observed in comparison with healthy individuals; recognition of this lectin correlates with disease activity [61]. Antibodies with reduced fucosylation of their Fc region have a better interaction with the Fc gamma receptor IIIa (FcgRIIIa) [53,70].

Increased recognition of the Lens culinaris lectin, which is specific for the core fucosylated trimannose *N*-glycan, towards immunoglobulin G has been also reported in SLE patients [61]. The increased exposure of mannose residues in IgG facilitates the activation of FcγRIIIa and increases antibody-mediated cytotoxicity [53]. These mannose-exposed glycans have few fucose residues, which may also affect FcγRIIIa binding; in addition, mannose-binding lectin (MBL) can recognize these mannose-exposed glycans in IgG and activate the complement lectin pathway [53].

Endoglycosidase S is an enzyme that cleaves the *N*-glycans of IgG, but not those of IgM, exposing an *N*-acetylglucosamine (GlcNAc) from the glycosidic core and its branched fucose residue. Treatment of BXSB mice that developed a lupus-like disease with endoglycosidase S did not reduce anti-dsDNA (double-stranded) or antinuclear antibody levels, but increased mouse survival and improved blood urea levels [71]. The proposed mechanism is that treatment with endoglycosidase S reduces the affinity of antibodies towards FcγR, which could prevent activation by immune complexes [72].

In the case of SLE patients, using ultra-resolution liquid chromatography, it was found that they present a decrease in IgG galactosylation and sialylation; a decrease in the fucose nucleus and an increase in *N*-acetylglucosamine bisector was also observed. These glycosylation abnormalities were associated with pericarditis, proteinuria, and the presence of antinuclear antibodies [73].

Altered glycosylation in antibodies has also been linked to the development of lupus nephritis. The presence of fucose in the *N*-glycans of IgG induces podocyte damage. The proposed mechanism is that the recognition of antibodies through the Fc receptor and lectin-like receptors triggers signals that increase the expression of calcium/calmodulin kinase IV (CaMK4), which in turn blocks the expression of Nephrin, an essential protein in the maintenance of the glomerular slit diaphragm. Interestingly, the presence of galactose in the *N*-glycosylations of the antibodies reduces the expression of calcium/calmodulin kinase IV [60].

Glycosylation of IgG anti-dsDNA from SLE patients was different from the glycosylation of total immunoglobulin G. It should be mentioned that the levels of fucosylation, galactosylation, and sialylation of anti-dsDNAse IgG increased with disease activity [74].

#### 5.1.2. Glycosylation of Variable Regions

The wide variety of antibodies with different affinities is due to the rearrangement of the variable (V), diversity (D), and joining (J) gene segments, as well as somatic hypermutation and class-switch recombination. During somatic hypermutation, point mutations occur in the variable regions of the heavy and light chains of immunoglobulins. These mutations can generate consensus sequences for *N*-glycosylation. The glycans attached to these sequences are called Fab glycans. It is estimated that 15% of IgGs have *N*-glycosylations in the variable regions, mainly as a result of hypermutation [75,76]. The function of these *N*-glycans is to regulate the affinity and stability of the antigen–antibody bond, as well as to prolong the half-life of the antibodies [68,69,77].

Patients with rheumatoid arthritis [78] and those with primary Sjögren’s syndrome [79] have been reported to have an increased number of *N*-glycosylation sites acquired by hypermutation. In the case of SLE, a meta-analysis of IgG heavy chain variable region sequences was performed to determine the increase in acquired *N*-glycosylation sites compared with sequences from patients without autoimmune diseases. An increase in acquired *N*-glycosylation sites has been found [80]; nevertheless, another study conducted in SLE patients and patients with myasthenia gravis found no difference in the frequency and distribution of *N*-glycosylation sites or glycosylation of the variable region of IgG and IgA [76]. Therefore, the functional role of these glycosylations in autoimmune diseases continues to be studied.

**Figure 3:**
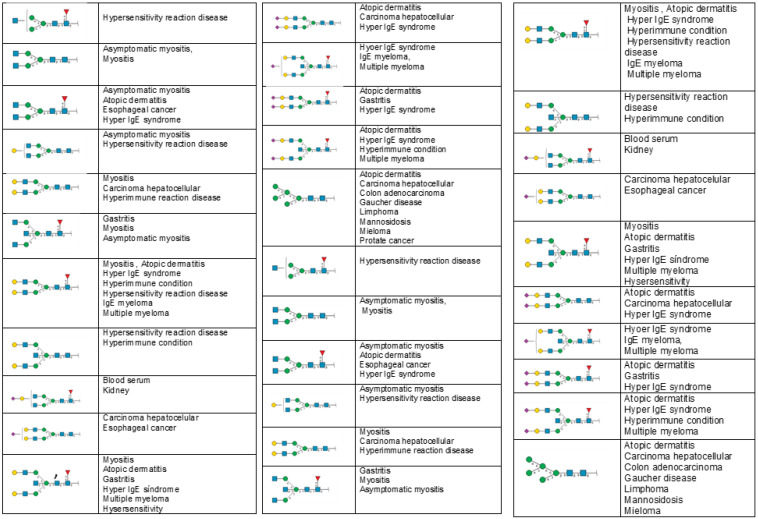
Altered glycans in the immunoglobulins of SLE patients and other diseases where they have been reported. Data were obtained from the GlyConnect Octopus database showing associations between proteins, diseases, and glycans.

In the GlyConnect Octopus database [81], twenty-two immunoglobulin G-linked glycans have been reported to be associated with SLE, and many of these glycans have been identified as related to other autoimmune diseases (Figure 3).

### 5.2. Alterations in the Glycosylation of the T Cells in SLE

It has been shown that T cells from SLE patients present alterations in *O*-glycosylation [62] using the *Amaranthus leucocarpus* lectin (ALL), which recognizes core 1 in coactivating T cell receptors [82]. T cells from patients with active SLE had less recognition by ALL than those with inactive SLE, and expression of receptors recognized by ALL was inversely correlated with disease activity [62].

Alterations in the expression of core 1 have also been described in murine models of lupus. T cells from MLR-lpr mice show reduced recognition of *Maclura pomifera* lectin compared to T cells from MLR++ mice [58]. The *Maclura pomifera* lectin is a Tn antigen-specific lectin [83]. MLR-lpr mice develop the disease faster and with greater clinical manifestations than MLR++ mice (Richard and Gilkeson, 2018), and show decreased *O*-glycosylation of their T cells similar to that seen in patients with active SLE [62].

Another study reported increased expression of Tn antigen-type *O*-glycans in T cells from SLE patients using *Vicia villosa* lectin and monoclonal antibodies (CT1 and CT2) [57,84]. Increased expression of Tn antigen has functional and regulatory implications in T cells, as studies have shown that macrophage galactose-binding lectin (MGL) can recognize the Tn antigen expressed on glycoprotein CD45 [40]. MGL binding to CD45 reduces its phosphatase activity and inhibits various signaling pathways, resulting in decreased secretion of proinflammatory cytokines, decreased proliferation, and induction of apoptosis [85].

Other lectins that recognize *O*-glycans, such as the *Arachis hypogaea* lectin or *Peanut Agglutinin* (PNA) and the *Artocarpus integrifolia* lectin (Jacalin), have not shown alterations in *O*-glycosylation in T cells from SLE patients [62]. These discrepancies may be due to the type of lectin used, since despite recognizing the same sugar, their affinity is variable and they recognize sugars present in different proteins [86,87].

For *N*-glycosylation, mice deficient in β-1,6 *N*-acetylglucosaminyltransferase V (Mgat5) develop autoimmune renal disease and are more susceptible to experimental autoimmune encephalomyelitis [88]. This is because Mgat5 deficiency prevents the addition of *N*-acetyllactosamine, the galectin 3 ligand, which mediates activation and survival functions in immune system cells; the reduced interaction of galectin 3 with the TCR lowers the activation threshold of T cells [88,89].

An increased fucosylation of CD4+ T cells has been described in SLE patients [90]. *N*-glycan fucosylation regulates T cell activation by affecting the interaction between MHC II and the TCR, and attenuating signaling through the TCR [91]; this could contribute to the active state of T cells in SLE.

Regarding sialylation, activated T cells from SLE patients have increased expression of ST6 β-galactosamine α-2,6-sialyltransferase 1 (ST6GAL1), the enzyme responsible for binding sialic acid at the α-2,6 bond galactose from *O*- and *N*-glycans; this increased expression of sialic acid prevents the binding of galectin 1 to its ligands [92]. Galectin 1 is a lectin that recognizes lactosamine and reduces the proliferation and activation of T cells in response to TCR stimuli [93]. In addition, galectin 1 has been related to apoptotic processes in activated T cells [94]. Since T cells from SLE patients showed reduced binding of galectin 1, these changes in ST6GAL1 expression could favor activation and reduce apoptosis of T cells.

We found no differences in the expression of α-2-3 and α-2-6 binding sialic acid in T cells from SLE patients using *Maackia amurensis* and *Sambucus Nigra* lectins [62]. However, lectins recognize sialic acid in a large number of proteins and are not suitable for evaluating changes in specific proteins, thus further studies are required to clarify whether there are alterations in the sialylation pattern in specific proteins.

In the regulatory T cells subpopulation, enzymatic removal of *N*-glycans has been reported to reduce their suppressive capacity; high levels of *N*-glycans were associated with increased expression of molecules related to the suppressive activity of regulatory T cells such as GITR, PD-1, PD-L1, CD73, CTLA-4, and ICOS. In cocultivation experiments, regulatory T cells expressing *N*-glycan complexes had greater suppressive activity than overactivated T cells [95]. Since SLE patients have defects in regulatory T cells [96], the expression of *N*-glycans could be a factor that affects their function and should be evaluated in future studies.

Several alterations in B and T cell glycosylation have been described in SLE, and how these alterations can affect the immune system. These changes in glycosylation correlate with disease activity and clinical manifestations, thus they could be used as a biomarker.

## 6. Alterations in Cytoplasmic *O*-GlcNAcylation

*O*-linked *N*-acetylglucosamine (*O*-GlcNAcylation) is a post-translational modification in which the monosaccharide *N*-acetylglucosamine (GlcNA) is added to serine or threonine residues of cytoplasmic or nuclear proteins. This transfer is mediated by *O*-GlcNAc transferase (OGT) and can be removed by *O*-GlcNAcase (OGA). Unlike the other types of glycosylation previously discussed, *O*-GlcNAcylation does not occur in the endoplasmic reticulum or Golgi apparatus, but rather in the cytosol. The addition of this monosaccharide to proteins has effects on protein localization, stability, and interactions [97]. *O*-GlcNAcylation regulates immune system functions such as inflammatory and antiviral responses in macrophages [98], promotes activated neutrophils functions [99], and inhibits natural killer cell activity [97,100].

A decrease in *O*-GlcNAcylation and phosphorylation of E74-like factor 1 (ELF-1) has been observed in SLE patients. ELF-1 is a transcriptional factor that binds to the promoter of the gene that encodes the zeta (ζ) chain of the CD3 molecule, thereby increasing its transcription. For this interaction to occur, the ELF-1 factor must be glycosylated and phosphorylated, constituting the 98 KDa isoform. Decreased glycosylation and phosphorylation of ELF-1 cause a decrease in its binding to DNA and a decrease in CD3ζ expression [101].

The decrease in *O*-GlcNAcylation and phosphorylation of E74-like factor 1 (ELF-1) is consistent with decreased expression of CD3ζ mRNA and its protein in SLE patients. To compensate for CD3ζ deficiency, the T-cell receptor complex associates with the Fc receptor gamma chain (FcRγ), causing alterations in intracellular signaling pathways as well as increased phosphorylation of tyrosine residues, an increase in the concentration of cytoplasmic calcium, and low production of IL-2 [102,103].

## 7. Conclusions

Protein glycosylation is a post-translational modification that regulates several processes in the immune system; its alterations have been related to autoimmune diseases. It has been shown that the deficiency of the enzyme α-mannosidase II in murine models leads to the development of lupus-like disease; in addition, in a study in SLE patients, a deficiency in this same enzyme was observed. This decrease is related to an increase in the expression of *N*-glycans with high mannose content, which correlates with the activation of the disease.

Also, alterations in the glycosylation of B cells and T cells of SLE patients have been identified, which are related to alterations in activation, differentiation, survival, and effector functions. In the case of B cells, studies have focused mainly on immunoglobulins in which altered glycans have been characterized, some of which are present in other autoimmune diseases. In T cells, several activation-related receptors show alterations in glycosylation, and these are related to disease activity; for this reason, it has been proposed that they could be used as biomarkers of activity.

However, studies on glycosylation in SLE are scarce, especially in B cells, where more studies are needed to evaluate the glycosylation of membrane receptors. It would also be important to evaluate whether there are glycosylation changes in other cells of the immune system, such as macrophages and dendritic cells.

Most of the studies have been based on the characterization of glycations with antibodies, lectins, and spectrometry, or the evaluation of the expression of enzymes involved in the synthesis of glycans; however, it is necessary to evaluate whether there are changes in the cellular localization of glycosyltransferases and glycosidases, as well as the effect of modulating the glycosylation of certain proteins in murine models of lupus. Finally, further studies are required to assess whether there are alterations in cytoplasmic *O*-GlcNAcylation in cells from SLE patients.
